# Hydroquinine Possesses Antibacterial Activity, and at Half the MIC, Induces the Overexpression of RND-Type Efflux Pumps Using Multiplex Digital PCR in *Pseudomonas aeruginosa*

**DOI:** 10.3390/tropicalmed7080156

**Published:** 2022-07-30

**Authors:** Nontaporn Rattanachak, Sattaporn Weawsiangsang, Touchkanin Jongjitvimol, Robert A Baldock, Jirapas Jongjitwimol

**Affiliations:** 1 Biomedical Sciences Program, Faculty of Allied Health Sciences, Naresuan University, Phitsanulok 65000, Thailand; nontapornr63@nu.ac.th (N.R.); sattapornw63@nu.ac.th (S.W.); 2 Biology Program, Faculty of Science and Technology, Pibulsongkram Rajabhat University, Phitsanulok 65000, Thailand; touchkanin@psru.ac.th; 3 School of Pharmacy and Biomedical Sciences, Faculty of Science and Health, University of Portsmouth, Portsmouth PO1 2DT, UK; robert.baldock@port.ac.uk; 4 Department of Medical Technology, Faculty of Allied Health Sciences, Naresuan University, Phitsanulok 65000, Thailand; 5 Centre of Excellence in Biomaterials, Faculty of Science, Naresuan University, Phitsanulok 65000, Thailand

**Keywords:** antibacterial agent, anti-malarial agent, hydroquinine, multiplex digital polymerase chain reaction (mdPCR), *Pseudomonas aeruginosa*, RND-type efflux pumps

## Abstract

Hydroquinine is an organic compound that is closely related to quinine-derivative drugs and contains anti-malarial and anti-arrhythmia activities. It has been also found in abundance in some natural extracts that possess antibacterial properties. However, there is little evidence demonstrating the antibacterial effect of hydroquinine. Therefore, we aimed to investigate the antibacterial properties of hydroquinine using broth microdilution methods. In addition, we evaluated the transcriptional responses of *P.* *aeruginosa* to hydroquinine-induced stress using RNA sequencing with transcriptomic analysis and validated the results using PCR-based methods. The MIC and MBC values of hydroquinine against all eight bacterial strains investigated ranged from 650 to 2500 and from 1250 to 5000 µg/mL, respectively. Transcriptomic analysis demonstrated that RND efflux pump transcripts were overexpressed (4.90–9.47 Log_2_ fold change). Using mRT-dPCR and RT-qPCR, we identified that mRNA levels of *mexD* and *mexY* genes were overexpressed in response to just half the MIC of hydroquinine in *P. aeruginosa*. In conclusion, we uncover the antimicrobial potential of hydroquinine as well as identify changes in gene expression that may contribute to bacterial resistance. Further work will be required to explore the efficacy and potential use of hydroquinine in the clinic.

## 1. Introduction

In recent years, the emergence of multidrug-resistant (MDR) bacterial pathogens has become a public health concern in many countries [1,2]. MDR pathogens mainly cause opportunistic infections in hospitalized patients, who have therapeutic options [1]. *Pseudomonas aeruginosa*, a non-glucose fermentative Gram-negative bacterium, is one of the most common opportunistic MDR pathogens, causing both hospital-acquired and community-acquired infections, e.g., in patients with burn wounds, cystic fibrosis, skin infections, and pneumonia [2,3]. Moreover, it has been statistically reported that bloodstream infections with *P. aeruginosa* are associated with high mortality rates of between 30 and 50% [4]. In intensive care units, nosocomial pneumonia caused by *P. aeruginosa* showed mortality rates of up to 70% [5]. The MDR *P. aeruginosa* has a mortality rate of 15–30% in some geographical areas [6,7,8]. Multidrug resistance can occur through mechanisms such as the production of enzymes (e.g., aminoglycoside-modifying enzymes, beta-lactamases, AmpC cephalosporinase), the decrease in outer membrane permeability (e.g., downregulation of *oprD* gene), and the hyperproduction of efflux pumps [9].

One of the main efflux pumps in *P. aeruginosa* is the Resistance-Nodulation-Cell Division (RND) superfamily, which strongly influences antibiotic susceptibility [10,11]. The RND-type efflux pumps recognize and expel a wide range of antibiotics with different structures and active sites, resulting in resistance to one or more drugs in microorganisms [12,13]. The efflux pump systems not only remove antibiotics but also pump out other substances and chemicals [14,15]. The RND efflux pumps directly expel substrate molecules from the inner membrane to the external medium with the aid of the proton gradient [9]. *P. aeruginosa* possesses at least 12 RND efflux pump systems, many of which can transport the same antibiotic substrates [9]. However, there are at least three clinically important multidrug exporter (Mex) systems, namely MexAB-OprM, MexCD-OprJ, and MexXY-(OprA) pumps [16,17]. MexAB-OprM and MexXY-(OprA) efflux pumps are normally expressed at the basal levels in wild-type *P. aeruginosa*; however, these are inducible when exposed to antibiotic substrates. In contrast, the MexCD-OprJ efflux pump is not expressed in wild-type strains, but it may contribute to antibiotic resistance when expressed in MDR strains [9]. The RND efflux pump systems of *P. aeruginosa* are intrinsic and serve to protect the organism from harmful agents [17]. The expression of RND pumps can be induced when *P. aeruginosa* responds to external stress factors, such as reactive oxygen species [9,18]. Some chemicals or agents also induce RND efflux expression in *P. aeruginosa* cells, including certain antibiotics, cinnamaldehyde, and sodium hypochlorite [19,20,21]. Therefore, efflux pumps serve as an important protective mechanism against cellular stresses, antibiotics, and potentially harmful environmental factors.

**Figure 1:**
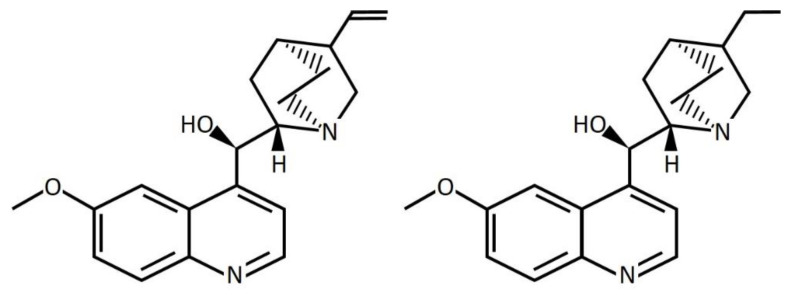
Chemical structures of quinine (**left**) and dihydroquinine (**right**).

Hydroquinine or dihydroquinine is an organic compound found in natural alkaloids (cinchona alkaloids) and is closely related to quinine (Figure 1) [22]. It has been well characterized as an anti-malarial agent effective against malaria in humans caused by *Plasmodium falciparum* and *Plasmodium knowlesi* [23,24], as well as a rodent malarial pathogen, e.g., *Plasmodium berghei* [25]. Recently, a study reported that hydroquinine could inhibit the tachyzoite growth of *Toxoplasma gondii*, which is a zoonotic parasite causing cerebral toxoplasmosis in humans [26]. According to our previous studies [27,28], hydroquinine was found in relative abundance in ethanolic crude extracts from the natural products of stingless bees. The extracts exhibited antibacterial activity against *Escherichia coli*, *Staphylococcus aureus,* and *P. aeruginosa,* as well as antifungal activity against *Candida albicans* and *C. parapsilosis* [27,28]. Interestingly, a quinine derivative, quinine sulfate, inhibited bacterial invasion and host-cell internalization into the skin [29,30]. However, there is little evidence about the antibacterial effect of the hydroquinine *in vitro* and the potential for the overexpression of RND efflux pumps in response to treatment. Therefore, this study aimed to investigate whether hydroquinine could inhibit any bacterial strains and whether the RND-type efflux pump systems would respond to hydroquinine treatment in *P. aeruginosa*. Here, we demonstrate the antibacterial effect of hydroquinine against several bacterial pathogens (reporting both the minimum inhibitory and minimum bactericidal concentrations, MIC and MBC, of hydroquinine). Furthermore in *P. aeruginosa*, we show the hydroquinine-induced upregulation of MexCD-OprJ and MexXY efflux pumps in response to sub-inhibitory hydroquinine concentrations.

## 2. Materials and Methods

### 2.1. Preparation of Hydroquinine Solution

Hydroquinine powder was purchased from Sigma-Aldrich and was used throughout this study. Hydroquinine solutions were dissolved with the appropriate volume of DMSO:Tween-80 (1:1) solution. The hydroquinine solution was accordingly labeled and closed away from the light source. All samples were freshly prepared for each experiment and syringe-filtered (through a 0.45 μm in pore size filter) before use in this study.

### 2.2. Bacterial Strains, Culture Conditions, and Inoculum Preparation

All reference strains used in this study were purchased from American Type Culture Collection (ATCC). There were eight bacterial strains, namely *S. aureus* ATCC29213, *S. aureus* ATCC25923, *P. aeruginosa* ATCC27853, *P. aeruginosa* ATCC BAA-2108, *E. coli* ATCC25922, *E. coli* ATCC2452, *Klebsiella pneumoniae* ATCC1705, and *Enterobacter cloacae* ATCC2341. All bacterial samples were cultured and maintained on tryptone soya agar (TSA; Oxoid, Basingstoke, UK) and incubated at 35 ± 2 °C for 18–24 h. In the case of subculturing, isolated colonies were re-streaked on the TSA plates. For inoculum preparation in each experiment, the bacterial suspension was grown until exponential phases, and the culture was adjusted to achieve turbidity using a densitometer (Biosan, Riga, Latvia), equivalent to a 0.5 McFarland standard.

### 2.3. Minimum Inhibitory Concentrations (MICs) and Minimum Bactericidal Concentrations (MBCs) of Hydroquinine

The antibacterial activity of hydroquinine was determined against all the bacterial strains used in this study using a broth microdilution method with a minor modification of the Clinical and Laboratory Standards Institute (CLSI) guideline M07-A9 [31]. Briefly, to determine the MIC values, 20,000 µg/mL of hydroquinine was freshly prepared by dissolving with 50% DMSO and Tween-80. The hydroquinine concentrations were serially diluted in Mueller–Hinton broth (MHB) to achieve a final volume of 100 µL and final concentrations of 0 (growth control), 19.5, 39.1, 78.1, 156.3, 312.5, 625, 1250, 2500, 5000, and 10,000 µg/mL on 96-well plates. For testing the vehicle control effect, 100 µL of only MHB containing 50% DMSO and Tween-80 were also included. Each 10 µL of the bacterial inoculum (1 × 10^8^ CFU/mL) was added to each well. Quality control (QC) tests were used to determine the precision and accuracy of the assays performed. In this case, a range of ciprofloxacin (CIP) concentrations from 0.0002 to 2 μg/mL were prepared to cover the known MIC values. *P. aeruginosa* ATCC27853 was used as a QC strain. Only MHB wells were performed as the sterility control without adding the bacteria tested. All 96-well plates were incubated at 35 ± 2 °C for 16–20 h. The MICs of hydroquinine were shown without bacterial growth by unaided eyes and were recorded. All results were acceptable when the MIC values of CIP against the QC strains must be within the QC ranges as shown in the CLSI document M100 [32]. All the tests were performed in triplicate experiments.

After the MIC results were read, the MBCs of hydroquinine were subsequently determined by pipetting 10 µL of each tested well from the culture to Mueller–Hinton agar (MHA) plates. The plates were incubated at 35 ± 2 °C for 24 h to observe the number of colonies. The least concentration without colonies grown on the MHA plates was recorded as the MBCs of hydroquinine (≥99.9% killing of the original inoculum [33]). All tests were performed in triplicate.

### 2.4. Time-Kill Curve of Hydroquinine

Either time-dependent or concentration-dependent bactericidal effect were observed by broth macrodilution time-kill assays. Briefly, a colony of *P. aeruginosa* was overnight cultured in 2 mL tryptone soya broth (TSB; Oxoid, Basingstoke, UK) with shaking at 100 rpm. The overnight culture was diluted in 100 mL fresh TSB and then incubated at 35 ± 2 °C for 3–6 h until an optical density of 0.4–0.6 was reached at 620 nm. The turbidity of the inoculum was again adjusted with fresh TSB to achieve turbidity of the 0.5 McFarland standard (1 × 10^8^ CFU/mL). Each 3 mL of the inoculum was added to each flask containing 30 mL MHB with different hydroquinine concentrations at MBC, MIC, ½ × MIC, and ¼ × MIC. The inoculum (3 mL) was also added to 30 mL of MHB as a growth control. All the flasks were incubated at 35 ± 2 °C with shaking at 100 rpm for 24 h. One milliliter of each culture was taken at each time interval of 0, 1, 2, 3, 4, 5, 6, 7, and 8 h. The samples were ten-fold serially diluted in sterilized phosphate-buffered saline (PBS) at dilutions of between 10^−1^ and 10^−10^. Each dilution was plated on MHA and incubated at 35 ± 2 °C for 24 h. All tests were performed in triplicate. The number of living cells of each flask was calculated in CFU/mL in order to calculate the percentage of killing compared to the growth control.

### 2.5. RNA Extraction and cDNA Synthesis

Total RNA samples were isolated using RNeasy Mini Kit (QIAGEN, Hilden, Germany). Briefly, a *P. aeruginosa* culture was harvested by centrifugation at 5000 rpm and 4 °C for 10 min. The pellet was resuspended with lysis buffer containing 1% ß-mercaptoethanol. The supernatant was transferred to a new microcentrifuge tube containing 70% ethanol, and the sample was then added into an RNeasy spin column placed into a collection tube. The tube was centrifuged at 14,000× *g* for 15 s. The RNeasy spin column was washed with washing buffer twice by centrifugation at 14,000× *g* for 60 s. To elute total RNA, 40 µL RNase-free water was added to the column. After centrifugation, the total RNA samples were checked for both quantity and quality using a Colibri Microvolume Spectrophotometer (Titertek-Berthold, Pforzheim, Germany). All RNA samples with good purity were kept at −80 °C for further analysis.

In the case of two-step RT-PCR to evaluate the gene expression, the complementary DNA (cDNA) synthesis step was performed using a QuantiNova Reverse Transcription Kit (QIAGEN, Hilden, Germany). To remove genomic DNA (gDNA), 1 μg of RNA was added to 2 µL of gDNA Removal Mix up to a 15 µL final volume with RNase-free water in a PCR tube. The tube was incubated at 45 °C for 2 min and then placed on ice. For cDNA synthesis, 4 µL of Reverse Transcription Mix and 1 µL of reverse transcription enzyme were added to the reaction tube. The reaction was incubated at the annealing step for 25 °C for 3 min, and reverse-transcription step was performed at 45 °C for 10 min, followed by inactivation of the reaction at 55 °C for 5 min. The concentration of cDNA synthesized was measured prior to downstream analysis.

### 2.6. RNA Sequencing and Transcriptomic Analysis

The 100 mL MHB containing *P. aeruginosa* ATCC27853 was incubated by shaking at 200 rpm at 35 ± 2 °C until reaching the mid-logarithmic phase (4–6 h). After that, 30 mL of the cultures were separated into two flasks. For the first flask, the hydroquinine solution was added to achieve the final concentration of 0.5 × MIC as the treated group. For a control, the same volume of vehicle control was added into the culture as a non-treated group. Both cultures were incubated at 200 rpm shaking, 35 ± 2 °C for 1 h. Then, the total RNA samples were isolated using an RNeasy Mini Kit (QIAGEN, Hilden, Germany) following the protocol described above. The integrity of RNA was verified by 1% agarose gel electrophoresis at 100 V for 40 min.

Transcriptomic analysis of the RNA samples (comparison of treated and non-treated groups) was then performed by Macrogen Inc. (Seoul, South Korea). Briefly, the RNA-Seq analysis included a quality check for raw sequencing data, reads mapping, expression quantification, differential expression genes (DEGs) analysis, and function enrichment analysis. After trimming the low-quality reads using FastP software version 0.19.7, passed filter reads were mapped to the *Pseudomonas aeruginosa* PAO1 genome reference using Bowtie2 version 2.3.4.1. Subsequently, transcript quantification and DEGs analysis were conducted using featureCounts and edgeR, respectively. The results of DEGs were summarized using the criteria of fold change ≥2 and false discovery rate (FDR) ≤ 0.05. Functional annotation analysis was also conducted using DAVID (https://david.ncifcrf.gov/ 2021 updated database, accessed on 11 May 2022).

### 2.7. Genomic DNA Extraction

Genomic DNA (gDNA) samples of all bacterial strains were isolated using a Genomic DNA Isolation Kit (Bio-Helix, New Taipei, Taiwan) following the manufacturer’s protocol. Briefly, each bacterial culture containing 10^9^ cells was transferred to a sterile microcentrifuge tube. The bacterial pellet was harvested by centrifuging at 12,000× *g* for one minute. The pelleted cells were resuspended with resuspension buffer, and the lysis buffer was added. After vortex mixing, the samples were incubated at 60 °C for 10 min. To degrade RNA, 5 µL of 10 mg/mL RNase A was then added and incubated at RT for 5 min. The protein-removal buffer was added to the sample tubes. The samples were centrifuged at 12,000× *g* for one minute. Each supernatant was transferred to a column that was placed in a 2 mL collection tube. After centrifugation, the column was washed with a washing buffer twice. To elute gDNA, 50 µL DNase-free water was added to the column. After centrifugation at 14,000× *g* for 2 min, the purity and concentration of the gDNA samples were checked using a Colibri Microvolume Spectrophotometer (Titertek-Berthold, Pforzheim, Germany). All gDNA samples with good purity were kept at −20 °C for further analysis.

### 2.8. Detection of RND-Type Efflux Pump Genes Using Multiplex Digital PCR (mdPCR)

In this study, the QIAcuity Probe PCR Kit (QIAGEN, Hilden, Germany) was used to carry out digital PCR (dPCR) using the QIAcuity Digital PCR system (QIAGEN, Hilden, Germany). All reactions were performed as multiplex digital PCR (mdPCR) in a 24-well platform (QIAcuity Nanoplate 26k 24-well, QIAGEN, Hilden, Germany). For mdPCR, 0.5 ng of the bacterial gDNA, 800 nM of each forward and reverse primer (Table 1), 400 nM of each probe (Table 1), and 10 µL of 4 × Probe PCR Master Mix were added and adjusted to a final volume of 40 µL with RNase-free water. The mixtures were then delivered onto the 24-well nanoplate, where the sample was sub-divided into approximately 26,000 partitions. The nanoplate was then sealed and transferred to the QIAcuity Digital PCR instrument containing a thermal cycler for PCR amplification using the following conditions: (i) PCR initial heat activation at 95 °C for 2 min, (ii) 40 cycles of denaturation at 95 °C for 15 s and combined annealing/extension at 59 °C for 30 s. The different fluorescent signals from each partition were then detected and calculated as positive and negative partitions. A non-template control (NTC) was included as a negative control.

### 2.9. Gene Expression Using Multiplex Reverse Transcription Digital PCR (mRT-dPCR)

To verify transcriptomic results, gene expression in *P. aeruginosa* ATCC27853, mRT-dPCR was carried out. All materials used for gene expression were synonymous with the mdPCR protocol mentioned in Section 2.8 except the type and number of templates. Briefly, 100 ng of *P. aeruginosa* cDNA samples were used instead of gDNA. The cDNA samples were synthesized from total RNA samples of *P. aeruginosa* treated and untreated with 0.5 × MIC hydroquinine for an hour. The thermal cycler conditions for amplification were the same with mdPCR. After the reaction was finished, the fluorescent signals were statistically calculated as positive and negative partitions. Non-template controls (NTC) were used as negative controls to calculate the appropriate threshold (cut-off) between positive and negative partitions. Data were expressed as the number of mRNA copies per microliter using Poisson statistics. Fold changes in mRNA were calculated as the average ratio of normalized mRNA copies per microliter in the hydroquinine-treated condition compared to the non-treated control.

### 2.10. Gene Expression Using Quantitative Reverse Transcription PCR (RT-qPCR)

To confirm the DEGs results of RND efflux pumps in both *P. aeruginosa* ATCC27853 and ATCC BAA-2108 at the different times of the hydroquinine treatment, the RT-qPCR reactions were performed in low-profile PCR tubes (Bio-Rad Laboratories, Hercules, CA, USA) and analyzed in a the LineGene 9600 Plus Real-Time PCR Detection System (Bioer Technology, Hangzhou, China). Briefly, each cDNA sample was reverse transcribed from the total RNA of both strains, which were treated and untreated with 0.5 × MIC hydroquinine for 1, 2, and 4 h treatment. Each reaction consisted of a 100 ng cDNA template, 800 nM of each forward and reverse primer (Table 1), 400 nM of each probe (Table 1), and 5 µL of 4 × Probe PCR Master Mix, which were added and adjusted to a 20 µL final volume with RNase-free water. The reactions were performed at 95 °C for 2 min, followed by 40 cycles of denaturation at 95 °C for 15 s and combined annealing/extension at 59 °C for 30 s. Each test was performed in triplicate together with a no-template control (NTC) in each run.

### 2.11. Statistical Analysis

The MIC and MBC values of hydroquinine from broth microdilution methods were presented as median values. Each plot of the killing curves was shown as mean ± 1 standard deviation. An independent student-t test was used to test for statistically significant mean differences between untreated and treated groups using IBM SPSS statistics version 23 (IBM Corp., Armonk, NY, USA). All graphs were created and analyzed using the GraphPad Prism version 8.2.0 (GraphPad Software, San Diego, CA, USA). For all analyses, the significant differences were considered with *p*-value < 0.05 representing statistical significance.

## 3. Results

### 3.1. Hydroquinine Showed MIC and MBC Values against the Bacteria Tested

We used the broth microdilution method to report MIC and MBC values (Table 2). It was found that hydroquinine inhibited all gram-positive and gram-negative bacteria tested in this study at different concentrations, showing MIC values between 650 and 2500 µg/mL. *E. coli* ATCC25922 was inhibited with the lowest MIC values (650 µg/mL), whereas *P. aeruginosa* ATCC27853 was inhibited with the highest MIC values (2500 µg/mL). We also demonstrated that hydroquinine killed all strains tested in this study at specific concentrations, showing MBC values between 1250 and 5000 µg/mL. It was noticeable that both *P. aeruginosa* strains were killed with the highest MBC values of 5000 µg/mL, which was approximately 2–4-fold higher than other strains assayed.

### 3.2. Time-Kill Curve of Hydroquinine against Both P. aeruginosa Strains

**Figure 2:**
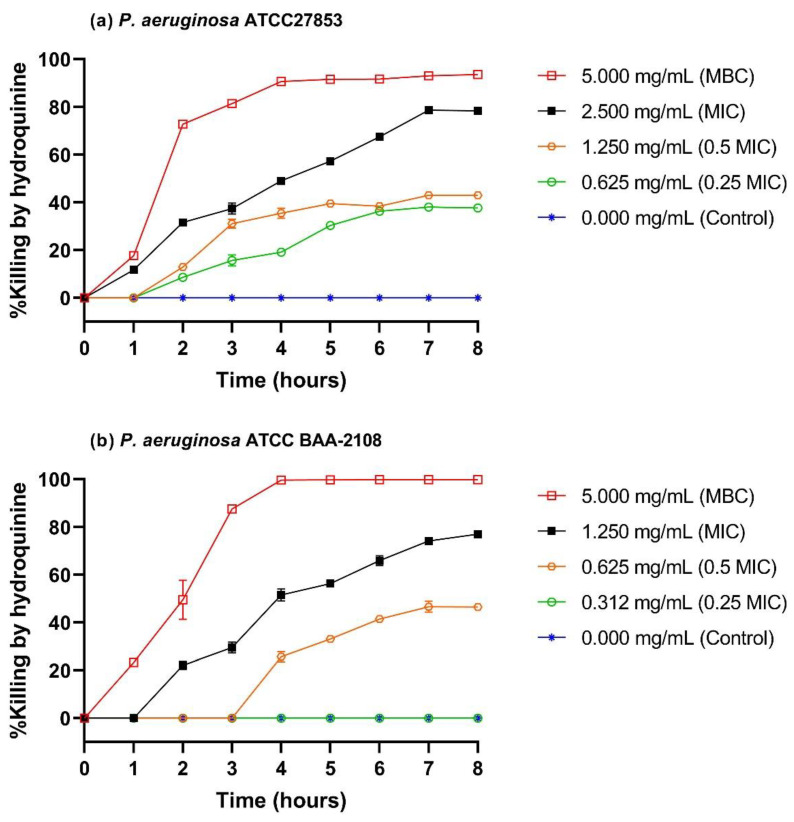
Time-kill curves of (**a**) drug-sensitive strain *P. aeruginosa* ATCC27853 and (**b**) multidrug-resistant strain *P. aeruginosa* ATCC BAA-2108 treated with and without indicated concentrations of hydroquinine at MBC, MIC, 0.5 × MIC, and 0.25 × MIC. MBC, minimum bactericidal concentration. MIC, minimum inhibitory concentration.

We examined the killing time of hydroquinine against both *P. aeruginosa* ATCC27853 and *P. aeruginosa* ATCC BAA-2108 using time-kill curve analysis. This showed that the number of both *P. aeruginosa* strains was close to 0% killing without hydroquinine (Figure 2a,b). After 4 h of treatment, the 0.5 × MIC of hydroquinine solution killed both strains in the region of 20–40%, whereas the MIC of hydroquinine showed the %killing at approximately 50% after 4 h. Ninety percent of each strain was killed after 4 h with the MBC values of hydroquinine. The %killing average of the hydroquinine-treated samples was found to be statistically different from that of the untreated group by 4 h in both *P. aeruginosa* strains (*p*-value < 0.05), except at the lowest dose tested (0.25 × MIC of hydroquinine) in *P. aeruginosa* ATCC BAA-2108 (Figure 2b).

### 3.3. Transcriptomic Analysis of P. aeruginosa ATCC27853 in the Stress Condition of Hydroquinine

We then screened the global transcriptomic profile of *P. aeruginosa* ATCC27853 treated with the 0.5 × MIC value (1250 µg/mL) of hydroquinine for an hour to examine the potential impact on gene expression. Interestingly, the DEGs results indicated that RND-type efflux pump genes, namely *mexC*, *mexD*, *oprJ*, *mexX,* and *mexY*, were highly overexpressed in response to the half MIC of hydroquinine (Table 3). The RND multidrug efflux membrane fusion protein MexC precursor was most upregulated by a 9.47 Log_2_-fold change, whereas the efflux transporter MexD and the efflux outer membrane protein OprJ precursor were upregulated by a 6.27 and 6.02 Log_2_-fold change, respectively. Moreover, the multidrug efflux membrane fusion protein precursor MexX and the efflux transporter MexY were upregulated by 5.26 and 4.90 Log_2_-fold changes, respectively.

### 3.4. All mexB, mexD, and mexY Genes Were Detected in Both P. aeruginosa Genomes Using mdPCR

All representative genes of *mexB*, *mexD,* and *mexY* were detected in the genomic DNA samples of *P. aeruginosa* ATCC27853 and *P. aeruginosa* ATCC BAA-2108 (Table 4). When mdPCR was used with the sets of specific primers and probes (Table 1), only positive partitions were present in both *P. aeruginosa* strains. It was evident that the chromosomes of *P. aeruginosa* ATCC27853 and *P. aeruginosa* ATCC BAA-2108 contained *mexB*, *mexD,* and *mexY* genes. However, these genes showed 0% positive partitions in the genomes of other strains, indicating that they did not contain *mexB*, *mexD,* and *mexY* genes.

### 3.5. Hydroquinine Induces the mRNA Levels of mexD and mexY in Both P. aeruginosa Strains

**Figure 3:**
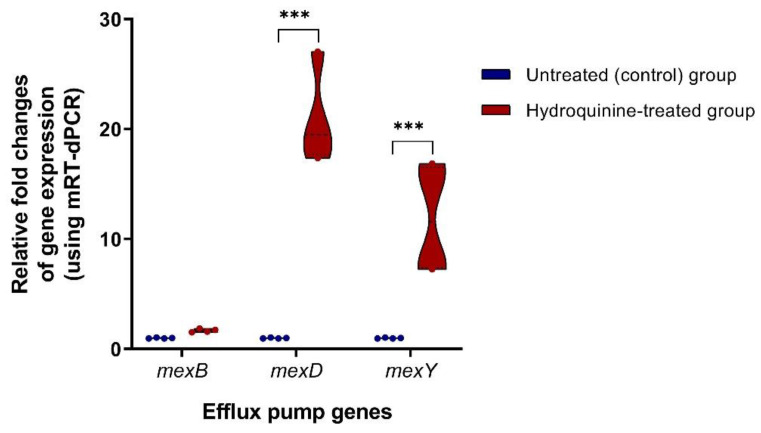
Expression levels of efflux pump genes, mexB, *mexD,* and *mexY*, in *P. aeruginosa* ATCC27853 using mRT-dPCR between untreated and treated with hydroquinine at 0.5 × MIC for 1 h. A symbol *** *p* < 0.001 was considered significant.

To determine whether hydroquinine induced the MexCD-OprJ and MexXY efflux pumps in *P. aeruginosa*, we used two PCR-based mRNA quantification methods. The first method was multiplex reverse transcription digital PCR (mRT-dPCR) to check the overexpression of MexCD-OprJ and MexXY efflux pumps in *P. aeruginosa* ATCC27853. Using this method, we found elevated expression levels of *mexD* (20.8 ± 4.31 fold) and *mexY* (11.8 ± 5.01 fold) after 1 h treatment of 0.5 × MIC hydroquinine. In contrast, *mexB* expression was not significantly up- or downregulated (1.6 ± 0.15 fold) (Figure 3).

**Figure 4:**
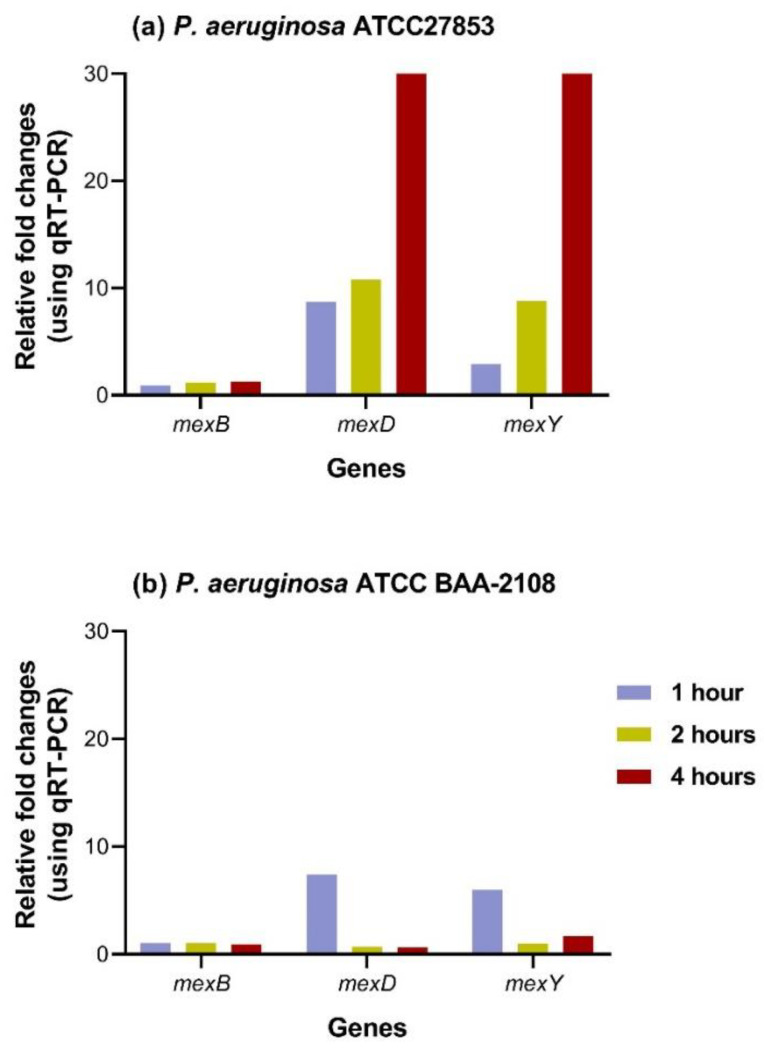
The mRNA expression of RND efflux pump genes in (**a**) *P. aeruginosa* ATCC27853 strain and (**b**) *P. aeruginosa* ATCC BAA-2108 strain treated with hydroquinine at the MIC value compared to the untreated conditions.

The second approach was reverse transcription quantification PCR (RT-qPCR) to check the overexpression of MexCD-OprJ and MexXY efflux pumps in both strains of *P. aeruginosa* at different times after 1, 2, and 4 h treatment. Consistently, after *P. aeruginosa* ATCC27853 was treated with hydroquinine for 4 h, the expression levels of *mexD* and *mexY* genes were increased between 8- and 30-fold and 3- and 30-fold, respectively (Figure 4a). After 60 min of treatment in *P. aeruginosa* ATCC BAA-2108, we found that the expression levels of *mexD* and *mexY* genes were increased approximately 7-fold and 6-fold, respectively (Figure 4b).

## 4. Discussion

In this study, we found that hydroquinine could inhibit and kill both Gram-positive and Gram-negative bacteria. This study firstly proved that hydroquinine inhibits *S. aureus* ATCC25923 and *S. aureus* ATCC29213 (Gram-positive bacteria), as well as *E. cloacae* ATCC2341, *E. coli* ATCC2452, *E. coli* ATCC25922, *K. pneumoniae* ATCC1705, *P. aeruginosa* ATCC27853, and *P. aeruginosa* ATCC BAA-2108 (Gram-negative bacteria), at different MIC values between 650 and 2500 µg/mL. We also determined that hydroquinine kills all these strains at MBC values between 1250 and 5000 µg/mL. This is consistent with previous reports that the crude extracts containing hydroquinine showed antibacterial properties against *Escherichia coli*, *Staphylococcus aureus,* and *P. aeruginosa* [27,28]. The reported MIC and MBC values of the crude extracts ranged between 6250 and >12,500 µg/mL against *S. aureus*, *E. coli,* and *P. aeruginosa* [27,28], whereas the MIC and MBC values of hydroquinine alone were 5–10-fold lower. In addition, the results of this study indicate that hydroquinine is likely to be a good candidate for development with better antibacterial activity than the quinine-derivative, quinine dihydrochloride (which has an MIC of 125 g/mL) [34]. Like hydroquinine, other quinine derivatives and quinine itself exhibit antibacterial activity against several pathogenic bacteria [35]. These findings highlight that hydroquinine is one of a number of pure bioactive compounds exhibiting antibacterial effects. However, the mode of action of hydroquinine as an antibacterial has yet to be uncovered and is of keen interest for future study. In addition, the side effects of hydroquinine should be investigated in future studies due to the limitations of our research. Few side effects on skin, eye, and respiratory irritation have been reported in the PubChem database [36]. However, the side effects of hydroquinine may be similar to those of quinine-based agents [37].

One point of note, however, is that the bactericidal concentrations of hydroquinine against both *P. aeruginosa* strains were 2–4-fold higher than those against the other strains tested (5000 µg/mL versus 1250–2500 µg/mL, respectively). We initially hypothesized that *P. aeruginosa* might possess a hydroquinine-resistance mechanism that promotes resistance to the killing effect of hydroquinine. We therefore focused on the cellular responses of both *P. aeruginosa* strains to hydroquinine. Using a time-kill assay to examine the antibacterial effectiveness, it was found that hydroquinine showed both bacteriostatic and bactericidal effects against each of the *P. aeruginosa* strains in a dose-dependent manner within 4–8 h after treatment. This is consistent with other quinine or alkaloid derivatives, which also exhibit bacteriostatic and bactericidal effects against microorganisms in a dose-dependent manner [38,39]. In the early phase of hydroquinine treatment, the number of living *P. aeruginosa* cells was quite stable, especially in the first hour following treatment. This may be due to the ability of the cells to adapt and respond to some environmental stresses (e.g., chemicals, low temperature, pH, etc.) in order to reprogram the transcriptomic profiles and then allow the cellular expression of protective functions [40]. Therefore, we wanted to identify any changes in *P. aeruginosa* ATCC27853 gene expression in response to hydroquinine treatment using transcriptomics. The treatment conditions were chosen based on the killing curve results with hydroquinine (Figure 2), with 0.5 × MIC not resulting in bacterial killing but still allowing the profiling of the transcriptomic changes after 1 h [40]. Transcriptomic analysis revealed that several genes associated with MexCD-OprJ and MexXY efflux pumps in *P. aeruginosa* ATCC27853 were overexpressed in response to the half MIC of hydroquinine (Table 3). As hypothesized, this may form part of a compensatory response in *P. aeruginosa* in which RND efflux pumps are upregulated to aid the removal of hydroquinine and promote survival.

To further validate whether hydroquinine induces the MexCD-OprJ and MexXY efflux pumps as well as MexAB-OprM in *P. aeruginosa*, a novel method, multiplex reverse transcription digital PCR (mRT-dPCR), was used to check for the presence of genes associated with MexAB-OprM, MexCD-OprJ, and MexXY efflux pumps, namely *mexB*, *mexD,* and *mexY*, respectively. All three genes (*mexB*, *mexD,* and *mexY*) were detected in the genomic DNA samples of *P. aeruginosa* ATCC27853 and *P. aeruginosa* ATCC BAA-2108 (Table 4). Using mdPCR, we were able to verify that the bacterial genomes of *P. aeruginosa* ATCC27853 and *P. aeruginosa* ATCC BAA-2108 contained *mexB*, *mexD,* and *mexY* genes. We then checked the overexpression of the RND-type efflux pumps in *P. aeruginosa* ATCC27853 using mRT-dPCR. This new method confirmed that the expression levels of *mexD* and *mexY* are increased by 20.8 ± 4.31 and 11.8 ± 5.01 fold after 1 h treatment with 0.5 × MIC hydroquinine, respectively. In contrast, the expression level of *mexB* remains relatively unchanged (1.6 ± 0.15 fold) (Figure 3). These data suggest that while hydroquinine induces the upregulation of the MexCD-OprJ and MexXY efflux pumps in *P. aeruginosa* ATCC27853 strain, the MexAB-OprM efflux pump is not upregulated. Unfortunately, due to technical limitations, it was not possible to verify the overexpression of the RND-type efflux pumps using mRT-dPCR in *P. aeruginosa* ATCC2018.

However, reverse transcription quantification PCR (RT-qPCR) was also performed to reproducibly validate the overexpression of MexCD-OprJ and MexXY efflux pumps in both strains of *P. aeruginosa* at 1, 2, and 4 h after hydroquinone treatment. Consistently, after *P. aeruginosa* ATCC27853 was again treated with 0.5 × hydroquinine for 1, 2, and 4 h, the expression levels of *mexD* and *mexY* genes were still increased by 8–30- and 3–30-fold, respectively (Figure 4a). After 60 min of treatment with hydroquinine in *P. aeruginosa* ATCC BAA-2108, the expression levels of *mexD* and *mexY* genes were increased by approximately 7-fold and 6-fold, respectively (Figure 4b). This consistency confirms that hydroquinine induces the mRNA levels of *mexD* and *mexY* in both *P. aeruginosa* strains. It further suggests that *P. aeruginosa* induces a protective mechanism by upregulating MexCD-OprJ and MexXY efflux pumps to avert cellular stress caused by hydroquinine. The RND-type efflux pump system is a tripartite complex, which comprises an inner membrane transporter (e.g., MexB, MexD, MexY), a periplasmic fusion protein (e.g., MexA, MexC, MexX), and an outer membrane channel (e.g., OprM, OprJ) [9]. Normally, these three components work together to pump substances out of the cells using the proton-motive force [9,41]. The inner membrane transporter proteins bind to specific substrates (including drugs) to facilitate their transport across the membrane, which is dependent on the proton gradient across the membrane (ΔpH). The proton gradient provides the driving force for this transport. The outer membrane protein facilitates the passage of the substrate across the outer membrane. The periplasmic membrane fusion protein connects both the inner and outer membrane proteins [14,15]. The substrate specificity of RND efflux pumps is provided by particular subunits, for example, amphiphilic molecules (e.g., MexB), hydrophobic solutes (e.g., MexD), and the hydrophilic polycationic aminoglycosides (e.g., MexY) [42]. Moreover, Morita et al. [43] reported that MexB binds negatively-charged substrates, whereas MexD and MexY do not. This may explain why the neutral charge and low water solubility of hydroquinine only affect the expression of *mexD* and *mexY* genes of *P. aeruginosa* after treatment. 

These findings suggest that hydroquinine has potentially useful antibacterial properties; however, it also induces specific RND-type efflux pumps. This supports the findings of previous studies, showing that individuals who receive antimalarial drugs, e.g., hydroquinine or quinine-based drugs, for treatment may also acquire a concomitant bacterial infection [29,30]. In these individuals, antibiotic treatments for active skin infections or other organ infections would be required [29,30]. Crucially, the mechanism of resistance through RND-type efflux pumps in *P. aeruginosa* infections should be screened in these concomitant cases to determine the potential role of efflux pump upregulation.

## 5. Conclusions

Here, we report that hydroquinine has antibacterial properties. It can inhibit and kill several strains of clinically important bacteria, namely *S. aureus*, *E. cloacae*, *E. coli*, *K. pneumoniae,* and, in particular, *P. aeruginosa*. Further research is required to uncover the mechanism of action; however, the findings of this study demonstrate that the low dose of hydroquinine treatment is sufficient to induce specific RND-type efflux pump systems in *Pseudomonas aeruginosa*, in particular the MexCD-OprJ and MexXY efflux pumps.

## Figures and Tables

**Table 1:** The sequences of primers and probes were used in this study.

Name	Oligonucleotide Sequences (5′ to 3′)	References
**Primers**		
*MexB F*	GATAGGCCCATTTTCGCGTGG	This study
*MexB R*	CGATCCCGTTCATCTGCTGC	This study
*MexD F*	TCATCAAGCGGCCGAACTTC	This study
*MexD R*	GGTGGCGGTGATGGTGATCTG	This study
*MexY F*	CGCAACTGACCCGCTACAAC	This study
*MexY R*	CGGACAGGCGTTCTTCGAAG	This study
*16s rRNA F*	CATGGCTCAGATTGAACGCTG	This study
*16s rRNA R*	GCTAATCCGACCTAGGCTCATC	This study
**Probes**		
*MexB*	(FAM) CGCCTTGGTGATCATGCTCGCG (BHQ1)	This study
*MexD*	(HEX) CTGGCCGGCCTGCTGGTCATTTC (BHQ1)	This study
*MexY*	(Texas Red) CGAAGCCATGCAGGCGATGGAGG (BHQ2)	This study
*16s rRNA*	(Cy5) CGAGCGGATGAAGGGAGCTTGCTC (BHQ2)	This study

**Table 2:** The minimum inhibitory and minimum bactericidal concentrations (MIC and MBC) values of hydroquinine against the microorganisms tested.

Microorganisms	MICs of Hydroquinine (µg/mL)	MBCs of Hydroquinine (µg/mL)	Vehicle Control ^1^ (%*v*/*v*)	MICs of CIP ^2^ (µg/mL)
**Gram positive bacteria**				
*S. aureus* ATCC25923	1250	1250	>25%	ND
*S. aureus* ATCC29213	1250	2500	>25%	0.5 (0.12–0.50)
**Gram negative bacteria**				
*E. cloacae* ATCC2341	1250	2500	>25%	ND
*E. coli* ATCC2452	1250	1250	>25%	ND
*E. coli* ATCC25922	625	1250	>25%	0.008 (0.004–0.016)
*K. pneumoniae* ATCC1705	1250	2500	>25%	ND
*P. aeruginosa* ATCC BAA-2108	1250	5000	>25%	ND
*P. aeruginosa* ATCC27853	2500	5000	>25%	0.5 (0.12–1.00)

^1^ Vehicle control (DMSO and Tween-80 solution) was used to dissolve hydroquinine. The MIC of >25% vehicle control means that the solvent did not influence the antibacterial results. ^2^ ND means “not determined” because no MIC QC ranges were provided in the CLSI document M100. The values in the blankets are the MIC QC ranges of the corresponding microorganisms shown in the CLSI document M100 [32].

**Table 3:** Differentially expressed genes (DEGs) associated with the RND-type efflux pumps as determined by transcriptome analysis.

Gene ID	Gene Name	Gene Description	Log_2_ FC ^1^	FDR ^2^	*p*-Value
NP_253289	*mexC*	Resistance-Nodulation-Cell Division (RND) multidrug efflux membrane fusion protein MexC precursor	9.47	2.56 × 10^−19^	4.79 × 10^−23^
NP_253288	*mexD*	Resistance-Nodulation-Cell Division (RND) multidrug efflux transporter MexD	6.27	3.83 × 10^−11^	3.59 × 10^−14^
NP_253287	*oprJ*	Multidrug efflux outer membrane protein OprJ precursor	6.02	2.90 × 10^−10^	3.80 × 10^−13^
NP_250709	*mexX*	Resistance-Nodulation-Cell Division (RND) multidrug efflux membrane fusion protein MexX precursor	5.26	2.47 × 10^−8^	5.55 × 10^−11^
NP_250708	*mexY*	Resistance-Nodulation-Cell Division (RND) multidrug efflux transporter MexY	4.90	9.60 × 10^−8^	2.88 × 10^−11^

^1^ Log_2_ FC = Log_2_ relative fold changes in gene expression of the hydroquinine conditions compared to the control; ^2^ FDR stands for false discovery rate with a statistical significance of *p*-value ≤ 0.05.

**Table 4:** Percentage of digital PCR (dPCR)-positive partitions of *mexB*, *mexD,* and *MexY* genes of each bacterial strain tested in this study.

Microorganisms/Sample	Positive Partitions (%)			Interpretation
	*mexB*	*mexD*	*mexY*	
*S. aureus* ATCC25923	0.0	0.0	0.0	Genes absent
*S. aureus* ATCC29213	0.0	0.0	0.0	Genes absent
*E. cloacae* ATCC2341	0.0	0.0	0.0	Genes absent
*E. coli* ATCC2452	0.0	0.0	0.0	Genes absent
*E. coli* ATCC25922	0.0	0.0	0.0	Genes absent
*K. pneumoniae* ATCC1705	0.0	0.0	0.0	Genes absent
*P. aeruginosa* ATCC BAA-2108	42.1	92.9	65.0	Genes present
*P. aeruginosa* ATCC27853	87.8	83.2	43.2	Genes present
Non-template control (NTC)	0.0	0.0	0.0	Genes absent

## Data Availability

Not applicable.
